# Epidemiological Surveillance and Mutational Pattern Analysis of Foot-and-Mouth Disease Outbreaks in Bangladesh during 2012–2021

**DOI:** 10.1155/2023/8896572

**Published:** 2023-08-30

**Authors:** Kazi Alamgir Hossain, Humaira Anjume, Masuda Akther, K. M. Mazharul Alam, Ashabul Yeamin, Salma Akter, M. Rafiul Islam, Munawar Sultana, M. Anwar Hossain

**Affiliations:** ^1^Department of Microbiology, University of Dhaka, Dhaka 1000, Bangladesh; ^2^Jashore University of Science and Technology, Jashore, Bangladesh; ^3^Department of Mathematics and Natural Sciences, BRAC University, Dhaka, Bangladesh; ^4^Department of Microbiology, Jahangirnagar University, Dhaka, Bangladesh

## Abstract

Foot-and-mouth disease (FMD) in cloven-hoofed animals is considered an economically devastating disease in endemic countries like Bangladesh, where the livestock sector contributes to a greater portion of the nation's economy. The causative agent of the disease, foot-and-mouth disease virus (FMDV), equipped with higher mutational frequency challenges the efficacy of the existing vaccine and control measures. This study, including 32 districts and 71 outbreaks to reveal epidemiological patterns and mutational trends of FMDV over the past 10 years (2012–2021), reported a 54.7% prevalence of FMD, with the majority of outbreaks occurring during the rainy season. Different risk factors such as age, gender, farming system, and vaccination status demonstrated a significant association with FMD cases which was confirmed by the *χ*^2^ test (*p* < 0.05). VP1 sequence analyses reported the predominance of serotype O (85%) over serotype A (11%) and serotype Asia 1 (4%). Bangladesh has foreseen the emergence of several novel FMDV strains during this decade. Novel sublineages, Ind-2001BD1 (Ind-2001e) and Ind-2001BD2, were reported under serotype O, the G-IX lineage of serotype Asia 1 emerged in 2018, and most recently in 2021, a new genotype named MYMBD21 under the lineage SA-2018 was detected for the first time in Bangladesh. Until now, Ind-2001e (Ind-2001BD1) sublineage under serotype O became the predominant sublineage in Bangladesh. From the mutational trend analysis, highly variable sites were observed at positions 138 and 140 within the G-H loop for serotype O. For serotype A and Asia 1, 45th and 44th residues within the B-C loop showed the highest amino acid variations, respectively. A changing mutational pattern among the 2019–2021 FMDV O and A isolates was also observed. The findings of the study would be crucial to understand the FMD situation and designing necessary preventive steps according to the progressive control pathway for FMD control in Bangladesh.

## 1. Introduction

Foot-and-mouth disease (FMD) contributes to huge economic losses each year in Bangladesh as well as many other endemic countries. The causative agent is an antigenically diverse RNA virus, foot-and-mouth disease virus (FMDV) which possesses a rapid transmission rate affecting cloven-hoofed animals such as cattle, buffalo, sheep, goats, and pigs, etc. [1, 2]. The mortality rate of FMD is relatively low and the morbidity rate can reach up to 100% [3, 4]. The occurrence of FMD is affected by different risk factors such as viral, host, and environmental factors [5].

FMDV is a member of the genus *Aphthovirus* and the family *Picornaviridae* [6–8]. The genome is about 8.5 kb and is surrounded by an icosahedral capsid [9]. Four structural proteins form the capsid, of which VP1, VP2, and VP3 are exposed outside, and VP4 is completely internal [4]. VP1 is the most variable among the other structural proteins consisting of three major antigenic sites: sites 1, 3, and 5 [10, 11]. The G-H loop, B-C loop and carboxy-terminus of VP1 conform these three major antigenic sites [12, 13].

The FMDV exists as seven immunologically distinct serotypes, namely A, O, C, Asia 1, and the Southern African Territories (SAT)-1, SAT-2, and SAT-3. Each serotype further contains multiple genotypes that are usually related to the geographical region of the disease occurrence [4, 7]. FMDV serotypes O, A, and Asia 1 are circulating in Bangladesh. Among them, serotype O was responsible for 82% of the outbreaks in Bangladesh [14]. Serotype C was not reported after 1990. Serotype A viruses were also reported in Bangladesh simultaneously with serotype O [14–16]. Circulation of FMDV Asia 1 serotype in Bangladesh is not consistent but sporadic [17].

A large FMD-susceptible livestock population of 24.7 million cattle, 26.7 million goats, 3.7 million sheep, and 1.5 million buffaloes exists in Bangladesh [18], and the occurrence of several outbreaks each year brings huge losses to the economy of Bangladesh. A study predicted that financial loss due to the FMD outbreak would be Tk. 188.57 billion or US$ 2.22 billion per year in Bangladesh [19].

The replication of the virus is erroneous and its polymerase lacks proofreading activity contributing to a wide variety of subpopulations [4, 7]. Previous infection or vaccination with one FMDV serotype does not confer cross-protective immunity to another. A high level of antigenic variation leads to the frequent emergence of novel FMDV strains within a serotype in Bangladesh that may reduce the efficacy of existing vaccines. In Bangladesh, the majority of FMDV vaccination program depends on imported vaccines, and the use of imported vaccines of heterologous virus strains often leads to incomplete protection or complete vaccine failure against local strains in Bangladesh. Moreover, this vaccine provides only short-time protection (∼6 months) [20, 21]. Under this circumstance, it becomes necessary to formulate FMD vaccines using circulating indigenous FMDV strains that would provide increased protection against the local virus. Again, the inadequate monitoring system, unrestricted transboundary movement of animals, and unplanned vaccination programs complicate the FMD situation in Bangladesh. All these facts necessitate constant epidemiological studies to keep track of the local strains and whether there is any emergence of newer genotypes of the virus which would be crucial to design a well-planned control strategy to prevent the disease realistically.

The current study was planned to conduct comprehensive surveillance to monitor the epidemiological situation of FMD in Bangladesh during a ten-year period from 2012 to 2021 to decipher the pattern of recent outbreaks and the effect of risk factors on the prevalence of FMD cases. The mutational trend in the VP1 sequence of circulating strains was also reported in this study. The study represents knowledge on currently circulating predominant strains as well as newly emerged FMDV strains in Bangladesh which would facilitate the selection of more appropriate FMD vaccine strains for developing more effective vaccines. This study also identified groups that are more susceptible to infection based on some risk factors such as age, gender, breed, farming system, and vaccination status of the animal, and the result would help the authorities to select which group should be prioritized more during the vaccination and control program. Therefore, the findings of the study would be valuable to design an effective control plan better suited for Bangladesh based on the prevailing FMDV situation in the country.

## 2. Materials and Methods

### 2.1. Sample Collection

From 2012 to 2021, a total of 481 tongue or foot epithelial tissue samples were collected from the infected lesion of FMD-suspected cattle, buffalo, and pigs covering 32 different districts of Bangladesh based on the notification of the clinical history from farmers and clinical findings within the animals. All the samples were collected by registered veterinary doctors without the use of anesthesia, following quite a safe and painless process. Sample collection was carried out following the protocol approved by the Animal Experimentation Ethical Review Committee, Faculty of Biological Sciences, University of Dhaka, with the permission of the herd owner. The ethical approval number for the study protocol is Ref: 66/Biol. Sci./2018-19; Date: November 14, 2018. A questionnaire was prepared (*Supplementary 1*) and filled during the collection of samples which describes the history of the patients. Samples were transported from the collection site to the laboratory at 4°C within 20 hr and stored at −80°C until processing and testing. All the samples were handled and processed in biosafety level 2 laboratory facilities.

### 2.2. RNA Extraction and cDNA Synthesis

The homogenization of tissue samples and total RNA extraction from tissue were performed in an automated Maxwell 16 system (Promega, USA) using the Maxwell 16 total RNA purification kit (Promega, USA) according to the manufacturer's instructions. Following extraction, the RNA was reverse transcribed into complementary DNA (cDNA) using the ImProm-IITM reverse transcription system (Promega, USA).

### 2.3. Polymerase Chain Reaction (PCR)-Based Amplification of VP1 and Sequencing of VP1

VP1-based PCR diagnostic assay was employed for the detection of FMDV-positive tissue samples. VP1 region of cDNA amplification was carried out using two sets of primers (VP1UF/NK61, 16F/NK61). The PCR reaction was performed using GoTaq 2× Hot Start Colorless Master Mix (Promega, USA) with either forward primer VP1UF (5′GTACTACRCSCAGTAC-3′) [22] or 16 F (5′-GAGAACTACGGWGGWGAGAC-3′) [16] and the reverse primer NK61 (5′-GACATGTCCTCCTGCATCTG-3′) [23]. The PCR products were run on 1.0% agarose gel with a 1 kb-DNA ladder (Promega, USA) for the visualization and detection of FMD-positive amplicons. Following detection, the FMDV-positive amplified PCR products were purified using the Wizard SV Gel and PCR Clean-Up System (Promega, USA) and were subjected to an automated cycle sequencing reaction using BigDye Terminator v3.1 Cycle Sequencing Kit (Applied Biosystems, USA) according to manufacturer's instructions with the same primers used in the PCR reaction. The VP1 coding sequence quality was analyzed in ABI Genetic Analyzer (Applied Biosystems, USA). Both forward and reverse sequences were assembled into a single contig using SeqMan version 7.0 (DNASTAR, Inc., Madison, WI, USA). The assembled sequences were compared with other sequences from GenBank using the basic local alignment search tool, BLAST [24], to reveal the identity of the isolated virus as well as their serotypes.

### 2.4. Epidemiological Analyses

The investigation of outbreaks in this study was based on the sample collected during 2012–2021 in the Microbial Genetics and Bioinformatics Laboratory, University of Dhaka [14, 25, 26]. We have followed a research-based data collection, and samples were collected upon communicating with outbreak information from the farmers as Bangladesh lacks systematic surveillance of FMD outbreaks. It is possible that not all outbreak information could be collected during the specified period of time. There could be a lack of samples from each and every incidence of outbreak cases. It should also be noted that the collection of the sample and reporting of the outbreak were interrupted by the pandemic situation of COVID-19 in Bangladesh during 2020–2021. Morbidity, mortality, and case fatality rates were calculated based on the population size (*n* = 3,580) included in this study. The influence of various risk factors such as season, age, gender, breed, and farming system on the occurrence of FMD cases was investigated and statistically verified by the chi-square (*χ*^2^) test at a 5% significance level using Statistical Package for Social Science, SPSS 26.0 for Windows (SPSS Inc., Chicago, IL, USA). The results of *χ*^2^ test were presented in *Supplementary 2*.

### 2.5. Phylogenetic Analysis

A total of 122 representative VP1-specific PCR products covering 71 outbreaks were sequenced from our laboratory from 2012 to 2021 and submitted into the NCBI database (https://www.ncbi.nlm.nih.gov/genbank/) [14, 16, 22, 25, 27–29].

Representative VP1 sequences of Bangladeshi isolates from 2012 to 2021 from our laboratory and also from reported sequences by other researchers in Bangladesh were included in the phylogenetic study to determine the genotype based on the clade formation in MEGA11 [30]. VP1 sequences of Bangladeshi isolates included in this study are listed in *Supplementary 2*.

The consensus VP1 coding sequences (complete 1D region) of local FMDV isolates were aligned using the ClustalW program [31] with the related gene sequences from GenBank. Phylogenetic Neighbor-Joining [32] trees were constructed (bootstrap replicates 1,000) based on the Kimura-2 parameter model [33]. A discrete Gamma distribution of a value of 1 was used to model evolutionary rate differences among sites. Fewer than 5% alignment gaps, missing data, and ambiguous bases were allowed at any position, as all the sequences were not completely aligned on the full range.

### 2.6. Analysis of VP1 Amino Acid Variations

The VP1 coding sequences of FMDV isolates reported in Bangladesh were used to analyze the overall variations among all FMDV serotype O, A, and Asia 1 viruses. The amino acid was translated based on the standard genetic code after codon-based alignment with MEGA11 software [30]. In the mutational pattern analysis, sequences under Ind-2001BD2 sublineage and also from MYMBD21 sublineage were excluded because sequences reported under both of these two sublineages had no amino acid variations.

## 3. Results

### 3.1. Epidemiological Investigation

**Figure 1:**
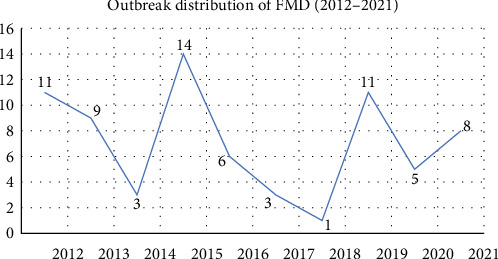
Distribution of FMD outbreaks from 2012 to 2021.

**Figure 2:**
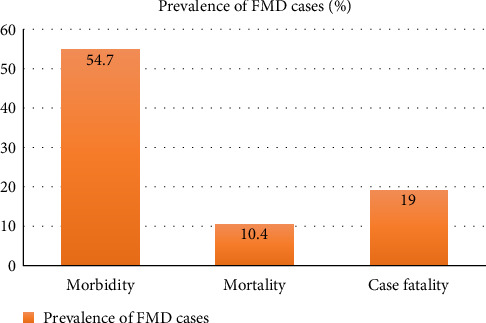
Morbidity, mortality, and case fatality rates of FMD.

Between 2012 and 2021, a total of 481 epithelial tissue samples representing 3,580 animal populations were collected from 32 districts which included 71 outbreaks (Figure 1), of which 230 samples were detected as FMDV positive in VP1-based PCR assay representing 1,960 population. These animal populations were included in the epidemiological studies. Decline in outbreaks in 2014, 2017, and 2018 might be due to a lack of systematic surveillance of FMD in Bangladesh and inability to include all outbreak information during the specified period of time. In the total population of animals (3,580), morbidity, mortality, and case fatality rates of FMD were 54.7% (1,960/3,580), 10.4% (372/3,580), and 19% (372/1,960), respectively (Figure 2).

#### 3.1.1. Risk Factor Analysis

**Figure 3:**
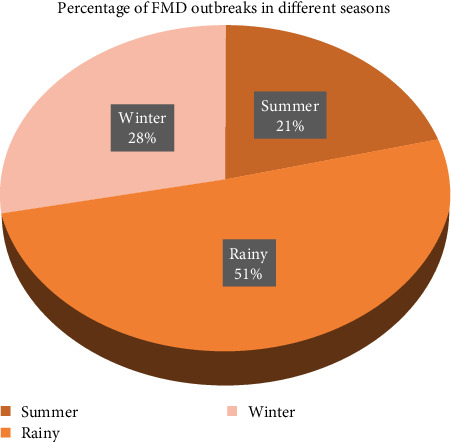
Percentage of FMD outbreaks in different seasons.

The occurrence of FMD outbreaks varied in different seasons. It was found that 51% (36/71) of the outbreaks occurred during the rainy season (July–October), followed by 28% (20/71) outbreaks during winter (November–February) and 21% (15/71) during summer (March–June) (Figure 3). In the *χ*^2^ test at 5% significance level, significant seasonal influence on the occurrence of the FMD outbreaks was observed where *p* < 0.001 was detected (*Supplementary 2*).

Various risk factors (age, gender, breed, farming system, and vaccination status) were considered in this study to observe the effect of these factors on the FMD morbidity rate.

**Figure 4:**
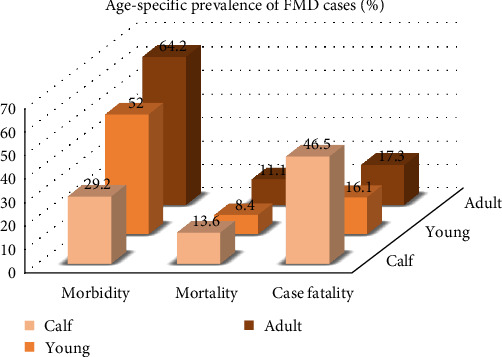
Age-specific prevalence of FMD cases.

Among them, in young cattle (>1 year before breeding) out of 1,365, morbidity, mortality, and case fatality rates were 51.9% (709), 8.4% (114), and 16.1%, respectively, while in adult population (1,728), the rates were 64.2% (1,109/1,728), 11.1% (192/1,728), and 17.3% (192/1,109), respectively. In other age groups (calves up to 1 year) out of 487, the morbidity rate was 29.2% (142), the mortality rate was 13.6% (66), and the case fatality rate was 46.5% (Figure 4). Morbidity was higher in adult populations, whereas mortality and fatality were higher in calves. *P* value was less than 0.05 (*p* < 0.001) in the *χ*^2^ test confirming a significant association of age and FMD prevalence (*Supplementary 2*).

**Figure 5:**
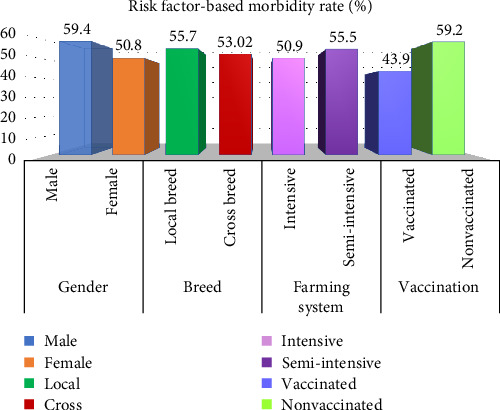
Risk factor (gender, breed, farming system, and vaccination) specific morbidity rates of FMD.

The morbidity rate of FMD was higher in male animals, 59.4% (970/1,632), than in females, 50.8% (990/1,948), where the *p*-value was <0.001 in the *χ*^2^ test (*Supplementary 2*). Local breed cattle showed 55.7% (1,267/2,273) morbidity and cross-breed cattle were 53% (693/1,307) susceptible to infection. The breed of animal did not have a significant association with the FMD cases, as *p*-value was greater than 0.05 in the *χ*^2^ test (*Supplementary 2*). The susceptibility rate of cattle reared under an intensive farming system was 50.9% (288/566) which is lower than that of animals under a semi-intensive farming system at 55.5% (1,672/3,014). *p*-Value less than 0.05 showing a significant association of the farming system with FMD cases (*Supplementary 2*). FMD was recorded in 43.9% (457/1,041) of the vaccinated cattle and 59.2% (1,503/2,539) in nonvaccinated cattle (Figure 5). Vaccination reduced the FMD cases by 1.3 times significantly, as confirmed by the *χ*^2^ test (*p* < 0.001) (*Supplementary 2*).

#### 3.1.2. Distribution of FMD Serotypes over Years (2012–2021)

Among the 71 outbreaks, 85% (60 out of 71) of the outbreaks were caused by serotype O, which was the prevalent serotype, whereas serotype A and Asia 1 were responsible for 11% (8 out of 71) and 4% (4 out of 71) of the outbreaks, respectively.

**Figure 6:**
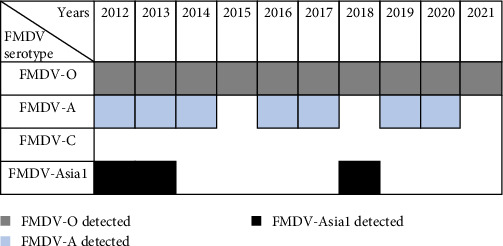
Occurrence of FMDV serotypes in Bangladesh (2012–2021).

From FMDV VP1 coding sequences reported in Bangladesh, it was revealed that serotype O, A, and Asia 1 was circulating during 2021–2021. Serotype O was detected each year from 2012 to 2021 [14, 26]. Serotype A was also present in the past 10 years but was not in circulation in 2015, 2018, and 2021 [16, 29]. Asia 1 was reported from 2012 to 2013 and then in 2018 [17, 22, 34]. No serotype C case was detected in Bangladesh (Figure 6) during the timespan.

### 3.2. Phylogenetic Analysis of FMDV Serotypes

Only representative FMDV VP1 sequences were considered in the phylogenetic study for the ease of data presentation from sequences reported from our laboratory [14, 16, 25, 27–29, 22] as well as from other researchers of Bangladesh [34, 35].

#### 3.2.1. FMDV Serotype O

**Figure 7:**
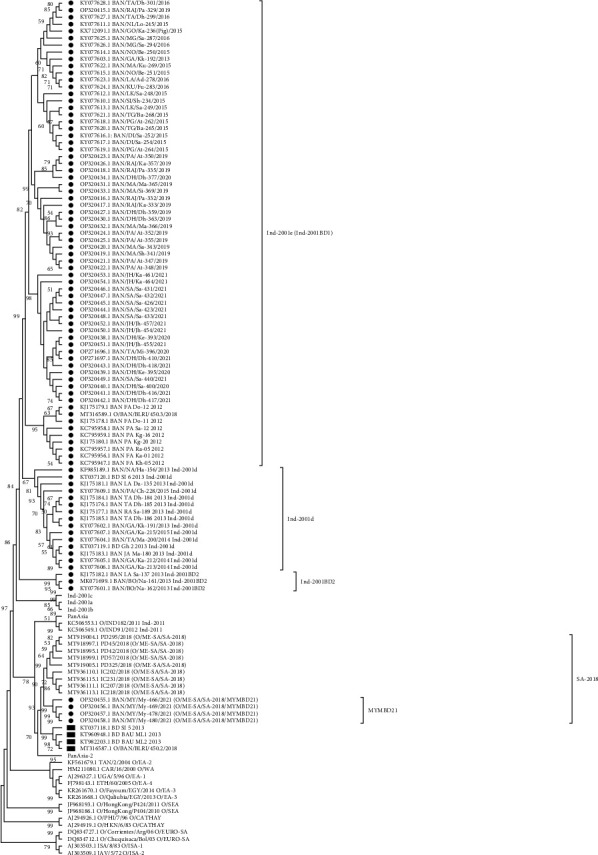
Phylogenetic reconstruction based on neighbor-joining method and Kimura-2 parameter model in MEGA11 showing subtypes of circulating serotype O in Bangladesh during 2012–2021. The isolates reported in Bangladesh are indicated with dot symbols, and unnamed isolates with square symbols. Some branches are compressed for better visualization.

Phylogenetic analysis demonstrated that from 2012 to 2021, two different lineages, Ind-2001 and SA-2018 lineages under serotype O, were reported to circulate in Bangladesh (Figure 7). Under the Ind-2001 lineage, Ind-2001d, Ind-2001BD1 (Ind-2001e), and Ind-2001BD2 sublineages were detected, among which only the Ind-2001BD1 sublineage was found in the last 5 years. Ind-2001BD1 was designated as Ind-2001e by the World Reference Laboratory for Foot-and-Mouth Disease, WRLFMD [36, 37]. The SA-2018 lineage was reported first in 2021 as a novel sublineage, MYMBD21 in Bangladesh [26]. In the phylogenetic tree, these isolates (OP320455.1-OP320458.1) called MYMBD21 showed a distinct clade with possible emergence from Indian SA-2018 isolates (Figure 7).

#### 3.2.2. FMDV Serotype A

**Figure 8:**
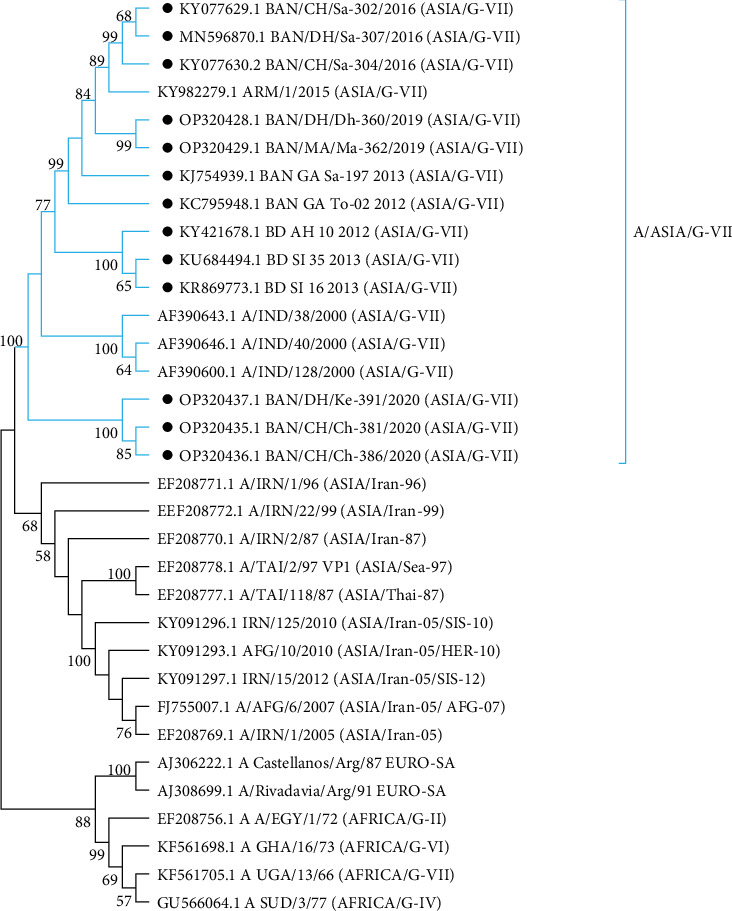
Phylogenetic reconstruction based on neighbor-joining method and Kimura-2 parameter model in MEGA11 showing topotype and lineage of FMDV serotype A circulating during 2012–2021. The sequences reported in Bangladesh are marked with black dots.

All the isolates under serotype A clustered within the ASIA topotype during 2012–2020. Among the lineages under this topotype, only the G-VII lineage was present in circulation (Figure 8).

#### 3.2.3. FMDV Serotype Asia 1

**Figure 9:**
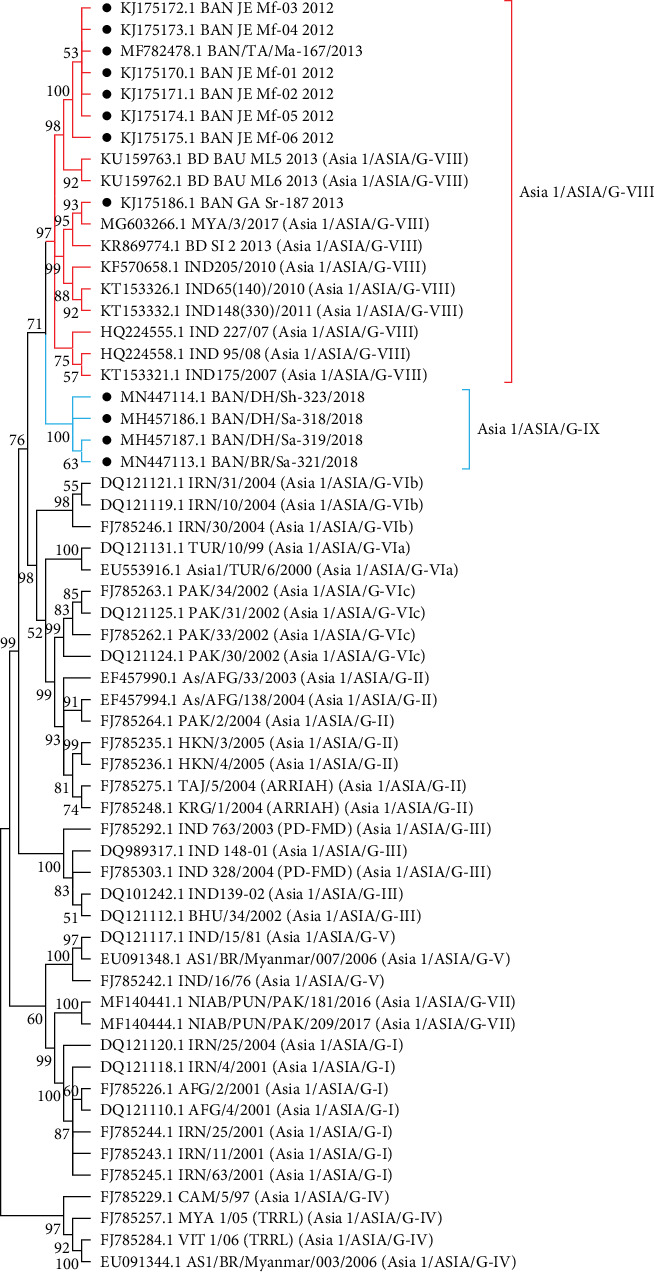
Phylogenetic reconstruction based on neighbor-joining method and Kimura-2 parameter model in MEGA11 showing lineages of serotype Asia 1 circulating in Bangladesh during 2012–2021. The sequences reported in Bangladesh are marked with black dots. Some branches are compressed for better visualization.

Phylogenetic reconstruction revealed that the G-VIII lineage was circulating during 2012–2013, and Asia 1 reemerged as a novel lineage G-IX (BD-18) in Bangladesh in 2018 [17] (Figure 9). No Asia 1 serotype was found since 2018. Sporadic circulation pattern of the Asia 1 serotype in Bangladesh was also observed in previous studies [17, 22].

**Figure 10:**
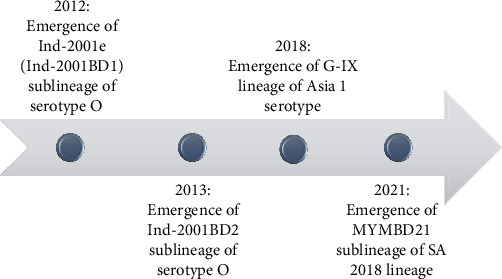
Emergence of novel strains of FMDV over the period of 10 years (2012–2021).

During 2012–2021, a few novel strains were introduced in FMDV circulation of Bangladesh, which is illustrated in Figure 10.

### 3.3. Analysis of VP1 Amino Acid Substitutions during 2012–2021

In Table 1, amino acid substitutions that occurred in the highly variable antigenic regions (B-C loop, G-H loop, and C-terminal) of FMDV VP1 during 2012–2021 timeline were listed. A list of all the mutations found in the VP1 from 2012 to 2021 was included in *Supplementary 2*. Only a few sequences of Ind-2001BD2 (3) and SA-2018/MYMBD21 (4) sublineages of serotype O were available that circulated only in a particular year. Hence, these lineages were excluded from the mutational study.

Mutations at the 52nd residue of the B-C loop, positions 135, 138, 139, 140, 155, 156, 158 of the G-H loop, and in the C-terminal 197, 200, 201, 204, 207, 212 positions were found only for the serotype O sequences. Unique mutations for serotype A were detected at positions 134, 143, 148, 190, 194, 196, and 209. In the case of Asia 1, N47S, T50V, M146L, and M211L were unique. Mutations at the 43rd and 48th residues were common to all three serotypes O, A, and Asia 1.

Table 2 represents the mutational pattern for serotypes O, A, and Asia 1 observed from 2012 to 2021. Here colored boxes (yellow for serotype O, blue for serotype A and black for serotype Asia 1) indicated the presence of a particular mutation in a given year, and white box represented the absence of the particular mutation.

Among serotype O isolates, more than two amino acid substitutions were found in positions: 43, 138, 140, 142, 197, 198, 201, and 204, among which 140th residues were more variable. Sublineage Ind-2001e (or Ind-2001BD1) sequences under serotype O had three mutations that persisted in the sequences from 2013 to 2021. These amino acid substitutions are—Q45K, N46D, and D197E. Those mutations might have become stable within the Ind-2001e sublineage. Sequences from Ind-2001e (or Ind-2001BD1) under serotype O varied mostly in C-termini and B-C loop from 2012 to 2018. In 2019, several unique substitutions: K41I, D52K, A140T, T142A, K204R, and L213F were detected. K135R occurred in 2020–2021 isolates of Ind-2001e. Ind-2001d was highly variable in 2013, with C-termini having the highest frequency of mutation. Ind-2001d showed diverse mutations throughout the years.

Serotype A showed mutations mostly in the B-C loop and G-H loop from 2012 to 2017. The frequency of mutation increased in the recent 2019–2020 samples, which had more mutations in the C-terminal region. Serotype A viruses from 2019 to 2020 retained some of the mutations from the viruses of 2012–2014. Sequences of 2019 retained I42L, V45T; 2020 sequences retained M190L, and S196L. V143T was seen in both the 2019 and 2020 viral sequences. Sequences from 2019 to 2020 had numerous unique mutations (N44D, V45L, S46N, N134S, R142H, E194K, Q198R) that were not found earlier. More than two amino acid exchanges were observed in residue-45, 46, 143, and 194.

Among serotype Asia 1 isolates, only the 44th residue had three amino acid substitutions. Within the Asia 1 serotype, A44T, E202K, V210M were specific for the G-VIII lineage. Several unique mutations were observed within the sequences of the G-IX lineage of serotype Asia 1: T43N, A44E, N47S, T50V, M146L, and M211L.

## 4. Discussion

In Bangladesh, FMD is endemic and considered a significant threat to the cattle industry. The time period of 2012–2021 was epidemiologically critical, showing changes in the circulation pattern of FMDV in Bangladesh with the emergence of multiple FMDV variants. The FMD cases included in the study were 19% fatal, with 10.4% mortality (Figure 2).

Occurrence of the highest percentage (51%) of FMD outbreaks during the rainy season might be due to relatively higher humidity and lower temperature that facilitated the survival of the virus, while higher temperature during summer might be responsible for the lower number of outbreaks. The effect of climate was also found in a previous study by Rahman et al. [41].

The morbidity rate was higher in the adult population than in calves and young cattle because older animals are more frequently get exposed to the multiple serotypes of FMDV over time at common grazing fields or markets via more contact with animals of other herds [42]. On the other hand, calves and young animals are mostly kept in home settings resulting in less contact with other animals. The relatively lower prevalence in younger animal groups (<2 years) might be due to the low frequency of contact with other herds as well as the presence of passive maternal immunity [42–45]. But mortality and fatality rates were higher in calves which might be due to the lack of immunity against multiple serotypes among calves, whereas adults become immune to multiple serotypes via frequent exposure (Figure 4). The reason behind more susceptibility in male animals (59.4%) than females (50.8%) might be due to more exposure of male cattle in the field settings for agricultural purposes compared to females. Working in the field increases the possibility of skin injury, and thus these cattle might easily become susceptible to infection whenever they come into close contact with other infected cattle [46, 47]. The higher susceptibility of cases was observed among local or indigenous breeds (55.7%) than that of crossbred cattle (53%) as in Bangladesh cross breed cattle are reared following more safety measures and regular vaccination in commercial farms for meat and milk production, whereas indigenous ones are kept in local households and extensively used for cultivation with no regular vaccination provided by the local farmers [46–48]. Under the intensive farming system, animals are kept in closed settings following proper animal health guidelines, whereas in semi-intensive farming, animals are allowed to graze on the field sometimes, which might have resulted in the higher prevalence of FMD in semi-intensive farms [46–49]. From the *χ*^2^ test at a 5% significance level, it was confirmed that season, age, gender, and farming system were significant factors for the occurrence of FMD (*p* < 0.05). But the association of breed with FMD cases was not found significant in the statistical (*χ*^2^) test (*p* > 0.05). Another important factor confirmed by the *χ*^2^ test was the vaccination status of the animal (*p* < 0.05). Vaccinated cattle had a lower risk of infection than nonvaccinated cattle.

The emergence of novel strains of FMDV within the time span of 10 years (2012–2021) is an epidemiologically significant event (Figure 10). In 2012, Ind-2001d was prevalent, but Ind-2001BD1 or Ind-2001e sublineage emerged in the same year [14], which replaced the Ind-2001d sublineage and later, in 2016, became the predominant FMDV sublineage in Bangladesh. Circulation of Ind-2001d was not detected after 2015. In the last few years (2016–2021), Ind-2001e (Ind-2001BD1) became the only circulating sublineage under the Ind-2001 lineage of FMDV in Bangladesh. Higher mutational frequency was detected within the VP1 of Ind-2001d viruses compared to the Ind-2001e sublineage in a previous study [14], which could be the possible reason for the disappearance of the Ind-2001d viruses from the field during the evolutionary selection process. On the other hand, less genetic diversity led to the selection of Ind-2001e over Ind-2001d as a dominant and single-circulating sublineage under the Ind-2001 lineage in circulation. The dominance of Ind-2001e over Ind-2001d and the gradual disappearance of the Ind-2001d lineage from circulation were supported by previous studies [14, 36, 50]. In 2013, another novel Ind-2001BD2 sublineage was detected, but its circulation did not continue in the subsequent years [14]. Under serotype Asia 1, G-VIII lineage was circulating during 2012–2013 after 1996, and then in 2018, Asia 1 serotype emerged as a novel lineage, G-IX (BD-18) in Bangladesh (Figure 9) [17]. Since 2018, no other cases of Asia 1 serotype were reported confirming the sporadic circulation pattern of this serotype. In 2021, a new lineage SA-2018 intruded in Bangladesh and was first reported from our Microbial Genetics and Bioinformatics Laboratory at the Department of Microbiology of the University of Dhaka [26]. This lineage was first reported from India in 2018 [51]. In VP1-based phylogeny, the isolates under SA-2018 lineage (OP320455.1-OP320458.1) formed a separate clade emerging from the Indian isolates indicating an evolutionary relationship with those Indian isolates (Figure 7), which might have resulted from unrestricted cattle movement or international trade at borders with India [21, 50]. The association of unregulated animal movement with the virus transmission across the country or among neighboring countries and the emergence of exotic FMDV strains were evident in previous studies [50–52]. In our previous study, these isolates were confirmed as a novel sublineage, MYMBD21, under SA-2018, lineage showing a 5%–6% nucleotide distance from the Indian isolates [26]. In the case of serotype A, only one lineage G-VII under ASIA topotype was detected in Bangladesh (Figure 8). Some isolates under serotype O were uncharacterized (BD_BAU_ML1_2013, BD_BAU_ML2_2013, BD_SI_5_2013, O/BAN/BLRI/450.2/2018), which require detailed VP1-based analyses (Figure 7) [35].

From the amino acid substitution analyses, it was evident that most of the changes occurred in the antigenic sites, the G-H loop, B-C loop, and C-terminal loop. The RGD motif containing Arg-Gly-Asp was found to be conserved in all serotypes as it is an important recognition element in integrin-dependent cell adhesion processes [4, 39, 53].

Many single amino acid substitutions were observed in the VP1 sequences of FMDV. Amino acid residues at 43–46, 48, 142, 198, 202, and 210 positions were variable in more than one serotype (Table 1). Amino acid substitutions K45Q, T142A of serotype O, E194K of serotype A, and E202K of serotype Asia 1 sequences were also apparent in previously conducted studies [54, 55–57]. Frequently reported exchanges were reported in amino acid residues—142, 194, and 210 in a previous study [4], and substitutions were also detected in those positions for Bangladeshi isolates included in this study (Table 1). Amino acid residue 142 is located within the immunodominant epitope, G-H loop adjacent to RGD motif, and substitutions at this site can alter the structural conformation of the G-H loop of VP1 depending on the type of amino acid exchange [58]. Again, amino acid variations at positions—134, 143, and 158 within G-H loop; positions—196, 198, 210, and 213 within C-terminal were detected in other studies [54, 55, 57, 59, 60–62, 63, 64], and these positions were also variable among Bangladeshi isolates in this study (Table 1). Substitutions at the 210th amino acid residue detected in serotype O and Asia 1 can cause failure in the VP1-2A product [4, 63, 65].

Amino acid exchanges in which a negatively charged amino acid was replaced by a positively charged amino acid had occurred in amino acid residues 52, 138, 194, 202. In residue 52, negatively charged aspartic acid (D) was substituted by positively charged lysine (K). Residues 138, 194, and 202 had common amino acid substitutions—negatively charged glutamic acid (E) was substituted by positively charged lysine (K). Another common case of amino acid exchange occurred in amino acid residue—44, 46, 140, where uncharged, polar asparagine (N) was substituted by polar, negatively charged aspartic acid (D) (Table 1). Position 194 was found as a variable site which is very close to 195–197 residues that form one of the walls of the heparan sulfate (HS) binding site. Introduction of positive charge by E194K mutation in serotype A can affect the HS receptor binding to the virus [4, 66].

Within the serotype O, Ind-2001d contains more genetically diverse viruses than the other type O sublineages found in Bangladesh [14]. Significant mutations S139G, N140A, P142T, and P158T were found between Ind-2001d and Ind-2001e sublineages which were significant for the G-H loop displacement between these two sublineages [14]. In Table 2, Ind-2001e isolates collected from 2019 to 2021 showed more variations in the G-H loop, whereas isolates from 2012 to 2018 mostly varied within B-C loop and G-H loop. Unique mutations in the G-H loop: K135R, A140T, and T142A during 2019–2021 circulation of Ind-2001e were detected, which suggest a changing mutational pattern that might affect the integrin receptor binding modulating the orientation of the G-H loop. Significant mutations at these sites might result in drastic changes in the antigenicity of the virus.

Serotype A isolates from 2019 to 2020 showed more variations in the B-C loop and C-terminus. Asparagine (N) is converted into negatively charged, aspartate (D) at the 44th residue located in the antigenic site 3 within the B-C loop and can affect the antigenicity [4, 66]. E194K (negative to positive charge conversion) and Q198R (uncharged to positive charge conversion) mutations at the C-terminal site can affect antigenic site 2 and thereby modulate the heparan binding site as these two positions are adjacent to one of the walls of the heparan binding site [4, 66, 67].

Within the G-VIII lineage of Asia 1 isolates, few mutations at position—44, 202, 210 were found, among which A44T demonstrated polarity change and E202K (glutamate, E to lysine, K) referred to negative charge conversion into positive charge. Among the unique mutations of G-IX lineage, charge and polarity shifts also occurred. Uncharged alanine (A) was converted into negatively charged glutamic acid (E) at the 44th residue, and changes in polarity were observed at position 50 where polar threonine (T) was substituted by nonpolar valine (V) in the B-C loop affecting the antigenic site. These mutations might be responsible for the lineage turnover from G-VIII to G-IX.

Apart from the mutational analysis presented in this study, further serological assays are required to confirm any changes in the antigenicity of the recently circulating FMDV strains.

Several significant mutations accumulated within the circulating FMDV virus in Bangladesh from 2012 to 2021. Some mutations at significant antigenic sites contributed to the frequent emergence of novel variants within 10-year period that challenge the effectiveness of the existing vaccines and available treatment options. For the effective control of the disease, regular monitoring of FMD outbreaks, risk factor analysis, and planning to imply effective measures should be given emphasis. Moreover, instead of using imported vaccines, local vaccines with indigenous virus strains should be administered. To counter the problem of vaccine failure, an effective trivalent vaccine was developed with three local circulating FMDVs O/BAN/TA/Dh-301/2016 (MK088170.1), A/BAN/CH/Sa-304/2016 (MK088171.1), and Asia1/BAN/DH/Sa-318/2018 (MH457186.1) from our Microbial Genetics and Bioinformatics Laboratory, Department of Microbiology, University of Dhaka. This vaccine satisfactorily elicited antibody titers in cattle and is expected to confer protection against all FMDVs in Bangladesh [20, 25]. This vaccine is not marketed yet, and the use of traditional imported vaccines is still going on causing incomplete protection, which, in turn, giving rise to the emergence of newer FMDV variants. For instance, a new sublineage, MYMBD21 of SA-2018 lineage emerged in Bangladesh in 2021. There was a considerable VP1 nucleotide (12%–13%) and amino acid sequence (5%) divergence of MYMBD21 isolate from both the current field vaccine strain, O/India/R2/75 (accession number: AF204276.1) and proposed local vaccine strain, BAN/TA/Dh-301/2016 (accession number: KY077628.1). Again, 3D analysis revealed the displacement of the critical antigenic site, G-H loop of VP1 of MYMBD21 strain from existing vaccine strains that raised the possibility of vaccine escape and confirmation of which requires further serological analyses [26]. However, the isolates (OP320455.1-OP320458.1) belonging to this novel variant were stored in the seed bank of Microbial Genetics and Bioinformatics Laboratory, Department of Microbiology, University of Dhaka for subsequent serological analysis to assess its suitability as vaccine strain and for developing an immediate vaccine, if necessary, in future. Thus, regular monitoring regarding FMD virus evolution must be carried out to mitigate the problem of vaccine failure.

This study reported overall FMD epidemiological pattern and risk factor-based analysis during 2012–2021, providing the track of circulating and emerging FMD strains, identifying high-risk groups, analyzing mutations and variable regions within the VP1 capsid protein. The findings of the study would be valuable for better understanding the current FMD situation, which would facilitate adopting effective preventive actions which will lead to achieve the implementation of the OIE/FAO prescribed Progressive Control Pathway for FMD road map in Bangladesh.

## 5. Conclusions

One country should have its own surveillance system to plan control strategies of any infectious disease based on the disease pattern prevailing in the particular region. But Bangladesh lacks constant epidemiological surveillance of FMD, which complicates the management of the disease. To investigate the FMD situation in Bangladesh, this study analyzed FMD outbreaks in 10 years timespan. Among different groups of animals, the older male cattle reared under semi-intensive farming system, indigenous and nonvaccinated cattle were found to be more prone to infection. These groups should be prioritized more while adopting any vaccination or other control strategies of FMD in Bangladesh. Detection of Ind-2001e sublineage of serotype O as the predominant genotype in recent years suggests its use as an indigenous vaccine strain. There was a recent evolution of a novel variant, MYMBD21 sublineage under SA-2018 lineage in Bangladesh that presented the possibility of vaccine escape and thus was stored in the seed bank for further serological analyses. Overall, this study provides prerequisite knowledge of FMD epidemiology in Bangladesh that would facilitate designing and implementing more appropriate control and preventive approaches, particularly for Bangladesh to fight against the highly infectious disease.

## Figures and Tables

**Table 1:** Amino acid substitutions at major antigenic regions of VP1 of FMDV serotypes during 2012–2021. Amino acid exchanges in different serotypes are separated with commas. Multiple exchanges for a single position are separated with forward slashes.

Region	FMDV serotype	Amino acid substitutions	Unique substitutions
B-C Loop (43–59) [14, 38]	O, A, Asia 1	T43A/I, G43V, T43N	
	A, Asia 1	N44D, A44T/E	A44E for Asia 1 (G-IX lineage)
	O, A	(Q45K; K45Q), V45A/T/L	K45Q for Ind-2001d sublineage
	O, A	N46D, S46G/N	
	Asia 1	N47S	For G-IX lineage
	O, A, Asia 1	I48V, T48I, I48T	I48T for Asia 1 (G-IX lineage)
	Asia 1	T50V	For Asia 1 (G-IX lineage)
	O	D52K	

G-H Loop (130–160) [14, 39]	A	N134S	
	O	K135R	
	O	E138G/A/K	
	O	S139G	For Ind-2001d sublineage
	O	(N140G/A/D; A140T)	N140G/A/D for Ind-2001d sublineage
	O, A	(P142T/A; T142A), R142H	P142T/A for Ind-2001d sublineage
	A	V143T/A	
	Asia 1	M146L	For Asia 1 (G-IX lineage)
	A	G148E	
	O	A155V	
	O	A156T	
	O	P158T	For Ind-2001d sublineage

C-Terminal (190–213) [14, 40]	A	M190L	
	A	E194K/D	
	A	S196L	
	O	D197E/G/S	For Ind-2001BD1 sublineage
	O, A	E198Q/D, Q198R	E198Q/D for Ind-2001d sublineage
	O	R200T	
	O	H201P/R	
	O, Asia 1	K202Q, E202K	E202K for Asia 1 (G-IX lineage)
	O	K204R/N	
	O	A207P	
	A	A209T	
	O, Asia 1	K210N, V210M	
	Asia 1	M211L	Asia 1 (G-IX lineage)
	O	L212F	
	O, A	L213F, L213P	

**Table 2:** Year-wise distribution of amino acid substitutions in VP1 antigenic regions of serotype O (yellow box), serotype A (blue box), and serotype Asia 1 (black box).

Antigenic region	Amino acid substitutions	2012	2013	2014	2015	2016	2017	2018	2019	2020	2021
**B-C loop**	K41I (O/Ind-2001e)								🟨		
	**I42L (A/G-VII)**	🟦	🟦	🟦					🟦		
	**G43V (A/G-VII)**					🟦			🟦		
	T43I (O/Ind-2001e)					🟨		🟨	🟨		
	**T43A (O/Ind-2001d)**		🟨								
	**T43N (Asia 1/G-IX)**							⬛			
	**N44D (A/G-VII)**									🟦	
	**A44T (Asia 1/G-VIII)**		⬛								
	**A44E (Asia 1/G-IX)**							⬛			
	**K45Q (O/Ind-2001d)**		🟨		🟨						
	**V45A (A/G-VII)**		🟦								
	**V45L (A/G-VII)**									🟦	
	**V45T (A/G-VII)**	🟦	🟦	🟦					🟦		
	Q45K (O/Ind-2001e)		🟨		🟨	🟨		🟨	🟨	🟨	🟨
	N46D (O/Ind-2001e)		🟨		🟨	🟨		🟨	🟨	🟨	🟨
	**S46G (A/G-VII)**					🟦					
	**S46N (A/G-VII)**									🟦	
	**N47S (Asia 1/G G-IX)**							⬛			
	**I48V (O/Ind-2001d)**		🟨								
	**T50V (Asia 1/G G-IX)**							⬛			
	D52K (O/Ind-2001e)								🟨		

**G-H loop**	**N134S (A/G-VII)**									🟦	
	K135R (O/Ind-2001e)									🟨	🟨
	**K135R (O/Ind-2001d)**				🟨						
	E138A (O/Ind-2001e)	🟨									
	E138K (O/Ind-2001e)					🟨			🟨		
	**E138G (O/Ind-2001d)**				🟨						
	**S139G (O/Ind-2001d)**		🟨								
	A140T (O/Ind-2001e)								🟨		
	**N140G (O/Ind-2001d)**		🟨		🟨						
	**N140A (O/Ind-2001d)**		🟨								
	**N140D (O/Ind-2001d)**		🟨		🟨						
	T142A (O/Ind-2001e)								🟨		
	**P142T (O/Ind-2001d)**		🟨		🟨						
	**R142H (A/G-VII)**								🟦	🟦	
	**V143T (A/G-VII)**	🟦	🟦	🟦					🟦	🟦	
	**V143A (A/G-VII)**					🟦					
	**G148E (A/G-VII)**	🟦									
	**M146L (Asia 1/G G-IX)**							⬛			
	**A155V (O/Ind-2001d)**		🟨								
	A156T (Ind-2001e)				🟨						
	**A156T (Ind-2001d)**		🟨								
	**P158T (Ind-2001d)**		🟨								

**C-terminal**	**M190L (A/G-VII)**	🟦	🟦	🟦						🟦	
	**E194D (A/G-VII)**	🟦									
	**E194K (A/G-VII)**								🟦		
	**S196L (A/G-VII)**		🟦							🟦	
	D197E (O/Ind-2001e)		🟨		🟨	🟨		🟨	🟨	🟨	🟨
	D197G (O/Ind-2001e)					🟨					
	**E198Q (O/Ind-2001d)**		🟨		🟨						
	**E198D (O/Ind-2001d)**		🟨	🟨							
	**Q198R (A/G-VII)**								🟦		
	**R200T (O/Ind-2001d)**		🟨								
	H201R (O/Ind-2001e)				🟨						
	**H201P (O/Ind-2001d)**		🟨								
	**K202Q (O/Ind-2001d)**		🟨								
	**E202K (Asia 1/G G-VIII)**		⬛								
	K204R (O/Ind-2001e)									🟨	
	**K204R (O/Ind-2001d)**				🟨						
	**K204N (O/Ind-2001d)**		🟨								
	**A207P (O/Ind-2001d)**		🟨								
	**A209T (A/G-VII)**	🟦									
	**V210M (Asia 1/G G-VIII)**		⬛								
	**K210N (O/Ind-2001d)**		🟨								
	**M211L (Asia 1/G G-IX)**							⬛			
	**L212F (Ind-2001d)**		🟨								
	L213F (O/Ind-2001e)									🟨	
	**L213P (A/G-VII)**		🟦								

## Data Availability

Data supporting the conclusions of the study can be found in the supplementary materials.

## Supplementary Materials

Supplementary 1 Model of the questionnaire used during sample collection. Ethical Clearance for the experiment (Ref: 66/Biol. Sci./2018-19; Date: November 14, 2018).

Supplementary 2 Chi-square test results for testing the significance of the association of risk factors with FMD cases calculated in SPSS Version 26.0. List of the FMDV VP1 sequences reported from Bangladesh during 2012–2021. Amino acid substitutions in VP1 of FMDV serotypes reported in Bangladesh during 2012–2021.
