# Supplementary material for: Epidemiological Surveillance and Mutational Pattern Analysis of Foot-and-Mouth Disease Outbreaks in Bangladesh during 2012–2021
Source: Transbound Emerg Dis. 2023 Aug 30;2023:8896572. doi: 10.1155/2023/8896572 (PMC12017146; doi:10.1155/2023/8896572)
Supplement: Supplementary 1 — Model of the questionnaire used during sample collection. Ethical Clearance for the experiment (Ref: 66/Biol. Sci./2018-19; Date: November 14, 2018). [file 8896572.f1.pdf]

Supplementary Figures

Data collection sheet

SL No.: Date:

Owner's name and address: ……………………………………………………………………

Patient's data:

Present condition:

Breed………………. Age……. Years Sex ……………

Clinical signs

- ☐ Excessive salivation
- ☐ Fever
- ☐ Lameness
- ☐ Vesicle formation
- ☐ Loss of milk production (milking cow)

Treatment: ☐ Yes ☐ No

If yes:

Vaccination: ☐ Yes ☐ No

If yes:

Previous condition:

Infection with FMDV: ☐ Yes ☐ No

Breed………………. Age ……..Years | Sex ……………

Number of death cases:

Treatment: ☐ Yes ☐ No

If yes: ……………………………………………………………………………

Vaccination: ☐ Yes ☐ No

If yes: ……………………………………………………………………………

Signature of collector

Supplementary Figure S1: Model of the questionnaire used during sample collection.

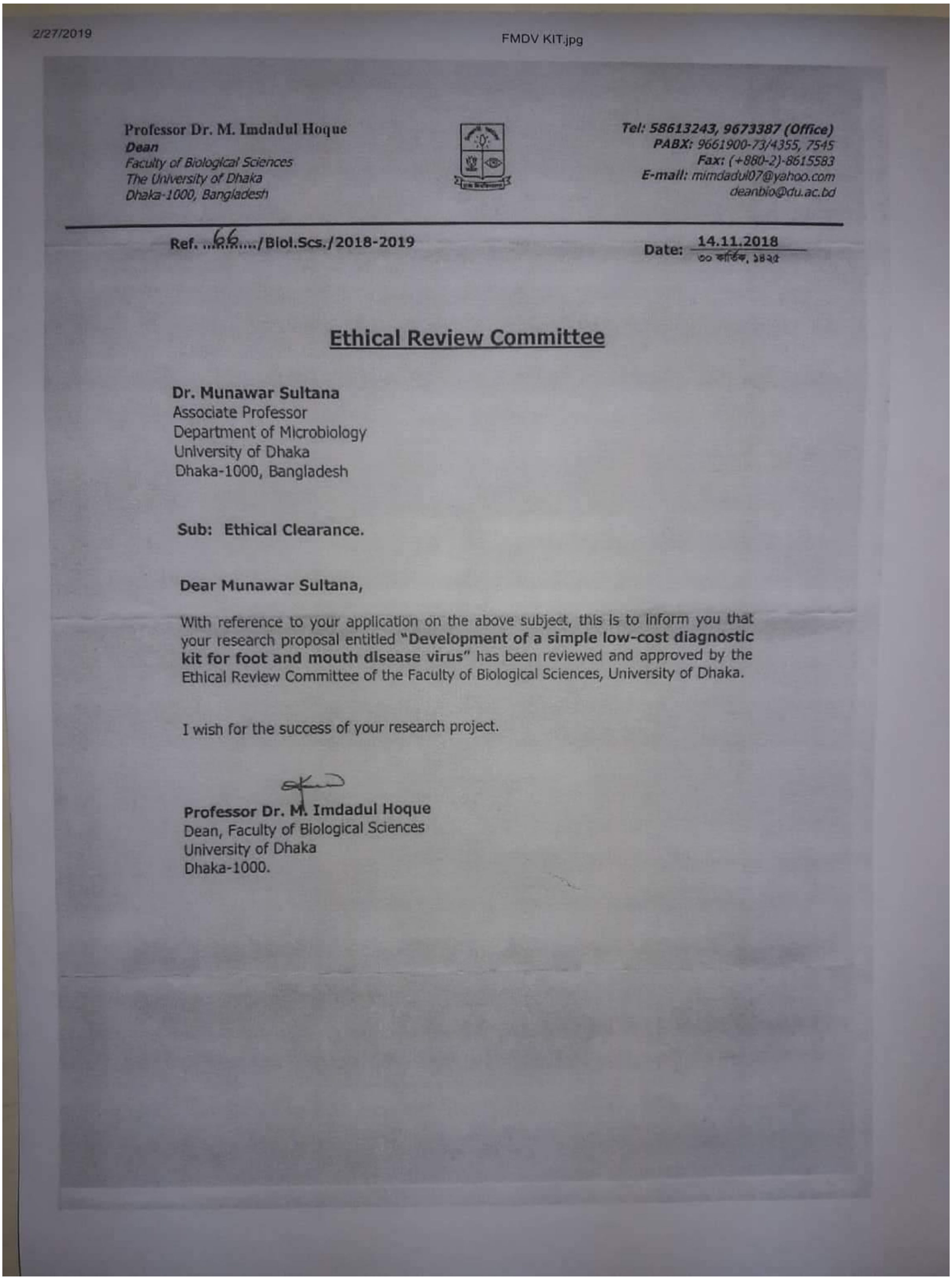

2/27/2019 FMDV KIT.jpg

Professor Dr. M. Imdadul Hoque
Dean
Faculty of Biological Sciences
The University of Dhaka
Dhaka-1000, Bangladesh

Tel:* *58613243, 9673387 (Office)
PABX: *9661900-73/4355, 7545*
Fax: *(+880-2)-8615583*
E-mail: *mimdadul07@yahoo.com*
deanbio@du.ac.bd

Ref. ...66...../Biol.Scs./2018-2019

Date: 14.11.2018
৩০ কার্তিক, ১৪২৫

Ethical Review Committee

Dr. Munawar Sultana
Associate Professor
Department of Microbiology
University of Dhaka
Dhaka-1000, Bangladesh

Sub: Ethical Clearance.

Dear Munawar Sultana,

With reference to your application on the above subject, this is to inform you that your research proposal entitled **"Development of a simple low-cost diagnostic kit for foot and mouth disease virus"** has been reviewed and approved by the Ethical Review Committee of the Faculty of Biological Sciences, University of Dhaka.

I wish for the success of your research project.

Professor Dr. M. Imdadul Hoque
Dean, Faculty of Biological Sciences
University of Dhaka
Dhaka-1000.

Supplementary Figure S2: Ethical Clearance for the experiment (Ref:66/Biol. Sci./2018-19; Date: 14-11-2018).
