# Supplementary material for: Epidemiological Surveillance and Mutational Pattern Analysis of Foot-and-Mouth Disease Outbreaks in Bangladesh during 2012–2021
Source: Transbound Emerg Dis. 2023 Aug 30;2023:8896572. doi: 10.1155/2023/8896572 (PMC12017146; doi:10.1155/2023/8896572)
Supplement: Supplementary 2 — Chi-square test results for testing the significance of the association of risk factors with FMD cases calculated in SPSS Version 26.0. List of the FMDV VP1 sequences reported from Bangladesh during 2012–2021. Amino acid substitutions in VP1 of FMDV serotypes reported in Bangladesh during 2012–2021. [file 8896572.f2.pdf]

Supplementary Tables

Chi-square test results:

Supplementary Table S1. Chi-square test results for testing the significance of the association of season with FMD cases calculated in SPSS Version 26.0

Chi-Square Tests (Season)

	Value	df	Asymptotic Significance (2-sided)
Pearson Chi-Square	15.254[a]	2	.000
Likelihood Ratio	15.098	2	.001
Linear-by-Linear Association	.789	1	.375
N of Valid Cases	213		

a. 0 cells (.0%) have expected count less than 5. The minimum expected count is 23.67.

Supplementary Table S2. Chi-square test results for testing the significance of the association of age with FMD cases calculated in SPSS Version 26.0

Chi-Square Tests (Age)

	Value	df	Asymptotic Significance (2-sided)
Pearson Chi-Square	195.092[a]	2	.000
Likelihood Ratio	197.847	2	.000
Linear-by-Linear Association	186.968	1	.000
N of Valid Cases	3580		

a. 0 cells (.0%) have expected count less than 5. The minimum expected count is 220.37.

Supplementary Table S3. Chi-square test results for testing the significance of the association of gender with FMD cases calculated in SPSS Version 26.0

Chi-Square Tests (Gender)

	Value	df	Asymptotic Significance (2-sided)	Exact Sig. (2-sided)	Exact Sig. (1-sided)
Pearson Chi-Square	26.603[a]	1	.000		
Continuity Correction[b]	26.256	1	.000		
Likelihood Ratio	26.664	1	.000		
Fisher's Exact Test				.000	.000
Linear-by-Linear Association	26.595	1	.000		
N of Valid Cases	3580				

a. 0 cells (.0%) have expected count less than 5. The minimum expected count is 738.50.

b. Computed only for a 2x2 table

Supplementary Table S4. Chi-square test results for testing the significance of the association of breed with FMD cases calculated in SPSS Version 26.0

Chi-Square Tests (Breed)

	Value	df	Asymptotic Significance (2-sided)	Exact Sig. (2-sided)	Exact Sig. (1-sided)
Pearson Chi-Square	2.477[a]	1	.116		
Continuity Correction[b]	2.368	1	.124		
Likelihood Ratio	2.475	1	.116		
Fisher's Exact Test				.117	.062
Linear-by-Linear Association	2.476	1	.116		
N of Valid Cases	3580				

a. 0 cells (.0%) have expected count less than 5. The minimum expected count is 591.44.

b. Computed only for a 2x2 table

Supplementary Table S5. Chi-square test results for testing the significance of the association of farming system with FMD cases calculated in SPSS Version 26.0

Chi-Square Tests (Farming system)

	Value	df	Asymptotic Significance (2-sided)	Exact Sig. (2-sided)	Exact Sig. (1-sided)
Pearson Chi-Square	4.054[a]	1	.044		
Continuity Correction[b]	3.871	1	.049		
Likelihood Ratio	4.041	1	.044		
Fisher's Exact Test				.048	.025
Linear-by-Linear Association	4.053	1	.044		
N of Valid Cases	3580				

a. 0 cells (.0%) have expected count less than 5. The minimum expected count is 256.12.

b. Computed only for a 2x2 table

Supplementary Table S6. Chi-square test results for testing the significance of the association of vaccination status with FMD cases calculated in SPSS Version 26.0

Chi-Square Tests (Vaccination Status)

	Value	df	Asymptotic Significance (2-sided)	Exact Sig. (2-sided)	Exact Sig. (1-sided)
Pearson Chi-Square	69.728[a]	1	.000		
Continuity Correction[b]	69.112	1	.000		
Likelihood Ratio	69.580	1	.000		
Fisher's Exact Test				.000	.000
Linear-by-Linear Association	69.708	1	.000		
N of Valid Cases	3580				

a. 0 cells (.0%) have expected count less than 5. The minimum expected count is 471.07.

b. Computed only for a 2x2 table

Supplementary Table S7. List of the FMDV VP1 sequences reported from Bangladesh during 2012-2021

Sequence ID	GenBank Accession No.	Serotype	Topotype	Lineage	Sublineage	Reference	Sample source
**BAN FA Ka-01 2012**	**KC795956.1**	**O**	**ME-SA**	**Ind-2001**	**Ind-2001e (Ind-2001BD1)**	**https://doi.org/10.1111/tbed.12834**	**MGBL lab, University of Dhaka**
**BAN FA Kh-05 2012**	**KC795947.1**	**O**	**ME-SA**	**Ind-2001**	**Ind-2001e (Ind-2001BD1)**	**https://doi.org/10.1111/tbed.12834**	**MGBL lab, University of Dhaka**
**BAN FA Do-11 2012**	**KJ175178.1**	**O**	**ME-SA**	**Ind-2001**	**Ind-2001e (Ind-2001BD1)**	**https://doi.org/10.1111/tbed.12834**	**MGBL lab, University of Dhaka**
**BAN FA Do-12 2012**	**KJ175179.1**	**O**	**ME-SA**	**Ind-2001**	**Ind-2001e (Ind-2001BD1)**	**https://doi.org/10.1111/tbed.12834**	**MGBL lab, University of Dhaka**
**BAN PA Ra-05 2012**	**KC795957.1**	**O**	**ME-SA**	**Ind-2001**	**Ind-2001e (Ind-2001BD1)**	**https://doi.org/10.1111/tbed.12113**	**MGBL lab, University of Dhaka**
**BAN PA Sa-12 2012**	**KC795958.1**	**O**	**ME-SA**	**Ind-2001**	**Ind-2001e (Ind-2001BD1)**	**https://doi.org/10.1111/tbed.12113**	**MGBL lab, University of Dhaka**
**BAN PA Kg-16 2012**	**KC795959.1**	**O**	**ME-SA**	**Ind-2001**	**Ind-2001e (Ind-2001BD1)**	**https://doi.org/10.1111/tbed.12113**	**MGBL lab, University of Dhaka**
**BAN PA Kg-20 2012**	**KJ175180.1**	**O**	**ME-SA**	**Ind-2001**	**Ind-2001e (Ind-2001BD1)**	**https://doi.org/10.1111/tbed.12834**	**MGBL lab, University of Dhaka**
**BAN/GA/Kk-192/2013**	**KY077603.1**	**O**	**ME-SA**	**Ind-2001**	**Ind-2001e (Ind-2001BD1)**	**https://doi.org/10.1111/tbed.12834**	**MGBL lab, University of Dhaka**
**BAN/SI/Sh-234/2015**	**KY077610.1**	**O**	**ME-SA**	**Ind-2001**	**Ind-2001e (Ind-2001BD1)**	**https://doi.org/10.1111/tbed.12834**	**MGBL lab, University of Dhaka**
**BAN/GO/Ka-236(Pig)/2015**	**KX712091.1**	**O**	**ME-SA**	**Ind-2001**	**Ind-2001e (Ind-2001BD1)**	**https://doi.org/10.1128/genomeA.01150-16**	**MGBL lab, University of Dhaka**
**BAN/NL/Lo-245/2015**	**KY077611.1**	**O**	**ME-SA**	**Ind-2001**	**Ind-2001e (Ind-**	**https://doi.org/10.1111/tbed.12834**	**MGBL lab, Universi**

					2001BD 1)		ty of Dhaka
BAN/LK/Sa-248/2015	KY077612.1	O	ME-SA	Ind-2001	Ind-2001e (Ind-2001BD 1)	https://doi.org/10.1111/tbed.12834	MGBL lab, University of Dhaka
BAN/LK/Sa-249/2015	KY077613.1	O	ME-SA	Ind-2001	Ind-2001e (Ind-2001BD 1)	https://doi.org/10.1111/tbed.12834	MGBL lab, University of Dhaka
BAN/NO/Be-250/2015	KY077614.1	O	ME-SA	Ind-2001	Ind-2001e (Ind-2001BD 1)	https://doi.org/10.1111/tbed.12834	MGBL lab, University of Dhaka
BAN/NO/Be-251/2015	KY077615.1	O	ME-SA	Ind-2001	Ind-2001e (Ind-2001BD 1)	https://doi.org/10.1111/tbed.12834	MGBL lab, University of Dhaka
BAN/DI/Sa-252/2015	KY077616.1	O	ME-SA	Ind-2001	Ind-2001e (Ind-2001BD 1)	https://doi.org/10.1111/tbed.12834	MGBL lab, University of Dhaka
BAN/DI/Sa-254/2015	KY077617.1	O	ME-SA	Ind-2001	Ind-2001e (Ind-2001BD 1)	https://doi.org/10.1111/tbed.12834	MGBL lab, University of Dhaka
BAN/PG/At-262/2015	KY077618.1	O	ME-SA	Ind-2001	Ind-2001e (Ind-2001BD 1)	https://doi.org/10.1111/tbed.12834	MGBL lab, University of Dhaka
BAN/PG/At-264/2015	KY077619.1	O	ME-SA	Ind-2001	Ind-2001e (Ind-2001BD 1)	https://doi.org/10.1111/tbed.12834	MGBL lab, University of Dhaka
BAN/TG/Ba-265/2015	KY077620.1	O	ME-SA	Ind-2001	Ind-2001e (Ind-2001BD 1)	https://doi.org/10.1111/tbed.12834	MGBL lab, University of Dhaka
BAN/TG/Ba-268/2015	KY077621.1	O	ME-SA	Ind-2001	Ind-2001e (Ind-2001BD 1)	https://doi.org/10.1111/tbed.12834	MGBL lab, University of Dhaka
BAN/MA/Ku-269/2015	KY077622.1	O	ME-SA	Ind-2001	Ind-2001e (Ind-2001BD 1)	https://doi.org/10.1111/tbed.12834	MGBL lab, University of Dhaka
BAN/LA/Ad-278/2016	KY077623.1	O	ME-SA	Ind-2001	Ind-2001e (Ind-2001BD 1)	https://doi.org/10.1111/tbed.12834	MGBL lab, University of Dhaka
BAN/KU/Fu-283/2016	KY077624.1	O	ME-SA	Ind-2001	Ind-2001e (Ind-	https://doi.org/10.1111/tbed.12834	MGBL lab, Universi

					2001BD 1)		ty of Dhaka
**BAN/MG/Sa-287/2016**	**KY077625.1**	**O**	**ME-SA**	**Ind-2001**	**Ind-2001e (Ind-2001BD 1)**	**https://doi.org/10.1111/tbed.12834**	**MGBL lab, University of Dhaka**
**BAN/MG/Sa-294/2016**	**KY077626.1**	**O**	**ME-SA**	**Ind-2001**	**Ind-2001e (Ind-2001BD 1)**	**https://doi.org/10.1111/tbed.12834**	**MGBL lab, University of Dhaka**
**BAN/TA/Dh-299/2016**	**KY077627.1**	**O**	**ME-SA**	**Ind-2001**	**Ind-2001e (Ind-2001BD 1)**	**https://doi.org/10.1111/tbed.12834**	**MGBL lab, University of Dhaka**
**BAN/TA/Dh-301/2016**	**MK088170.1**	**O**	**ME-SA**	**Ind-2001**	**Ind-2001e (Ind-2001BD 1)**	**https://doi.org/10.1111/tbed.12834**	**MGBL lab, University of Dhaka**
**BAN/RAJ/Pa-329/2019**	**OP320415.1**	**O**	**ME-SA**	**Ind-2001**	**Ind-2001e (Ind-2001BD 1)**	**https://doi.org/10.1038/s41598-023-36830-w**	**MGBL lab, University of Dhaka**
**BAN/RAJ/Pa-332/2019**	**OP320416.1**	**O**	**ME-SA**	**Ind-2001**	**Ind-2001e (Ind-2001BD 1)**	**https://doi.org/10.1038/s41598-023-36830-w**	**MGBL lab, University of Dhaka**
**BAN/RAJ/Ka-333/2019**	**OP320417.1**	**O**	**ME-SA**	**Ind-2001**	**Ind-2001e (Ind-2001BD 1)**	**https://doi.org/10.1038/s41598-023-36830-w**	**MGBL lab, University of Dhaka**
**BAN/RAJ/Pa-335/2019**	**OP320418.1**	**O**	**ME-SA**	**Ind-2001**	**Ind-2001e (Ind-2001BD 1)**	**https://doi.org/10.1038/s41598-023-36830-w**	**MGBL lab, University of Dhaka**
**BAN/MA/Sh-341/2019**	**OP320419.1**	**O**	**ME-SA**	**Ind-2001**	**Ind-2001e (Ind-2001BD 1)**	**https://doi.org/10.1038/s41598-023-36830-w**	**MGBL lab, University of Dhaka**
**BAN/MA/Sa-343/2019**	**OP320420.1**	**O**	**ME-SA**	**Ind-2001**	**Ind-2001e (Ind-2001BD 1)**	**https://doi.org/10.1038/s41598-023-36830-w**	**MGBL lab, University of Dhaka**
**BAN/PA/At-347/2019**	**OP320421.1**	**O**	**ME-SA**	**Ind-2001**	**Ind-2001e (Ind-2001BD 1)**	**https://doi.org/10.1038/s41598-023-36830-w**	**MGBL lab, University of Dhaka**
**BAN/PA/At-348/2019**	**OP320422.1**	**O**	**ME-SA**	**Ind-2001**	**Ind-2001e (Ind-2001BD 1)**	**https://doi.org/10.1038/s41598-023-36830-w**	**MGBL lab, University of Dhaka**
**BAN/PA/At-350/2019**	**OP320423.1**	**O**	**ME-SA**	**Ind-2001**	**Ind-2001e (Ind-**	**https://doi.org/10.1038/s41598-023-36830-w**	**MGBL lab, Universi**

					2001BD 1)		ty of Dhaka
**BAN/PA/At-352/2019**	**OP320424.1**	**O**	**ME-SA**	**Ind-2001**	**Ind-2001e (Ind-2001BD 1)**	**https://doi.org/10.1038/s41598-023-36830-w**	**MGBL lab, University of Dhaka**
**BAN/PA/At-355/2019**	**OP320425.1**	**O**	**ME-SA**	**Ind-2001**	**Ind-2001e (Ind-2001BD 1)**	**https://doi.org/10.1038/s41598-023-36830-w**	**MGBL lab, University of Dhaka**
**BAN/RAJ/Ka-357/2019**	**OP320426.1**	**O**	**ME-SA**	**Ind-2001**	**Ind-2001e (Ind-2001BD 1)**	**https://doi.org/10.1038/s41598-023-36830-w**	**MGBL lab, University of Dhaka**
**BAN/DH/Dh-359/2019**	**OP320427.1**	**O**	**ME-SA**	**Ind-2001**	**Ind-2001e (Ind-2001BD 1)**	**https://doi.org/10.1038/s41598-023-36830-w**	**MGBL lab, University of Dhaka**
**BAN/DH/Dh-363/2019**	**OP320430.1**	**O**	**ME-SA**	**Ind-2001**	**Ind-2001e (Ind-2001BD 1)**	**https://doi.org/10.1038/s41598-023-36830-w**	**MGBL lab, University of Dhaka**
**BAN/MA/Ma-365/2019**	**OP320431.1**	**O**	**ME-SA**	**Ind-2001**	**Ind-2001e (Ind-2001BD 1)**	**https://doi.org/10.1038/s41598-023-36830-w**	**MGBL lab, University of Dhaka**
**BAN/MA/Ma-366/2019**	**OP320432.1**	**O**	**ME-SA**	**Ind-2001**	**Ind-2001e (Ind-2001BD 1)**	**https://doi.org/10.1038/s41598-023-36830-w**	**MGBL lab, University of Dhaka**
**BAN/MA/Si-369/2019**	**OP320433.1**	**O**	**ME-SA**	**Ind-2001**	**Ind-2001e (Ind-2001BD 1)**	**https://doi.org/10.1038/s41598-023-36830-w**	**MGBL lab, University of Dhaka**
**BAN/DH/Dh-377/2020**	**OP320434.1**	**O**	**ME-SA**	**Ind-2001**	**Ind-2001e (Ind-2001BD 1)**	**https://doi.org/10.1038/s41598-023-36830-w**	**MGBL lab, University of Dhaka**
**BAN/DH/Ke-393/2020**	**OP320438.1**	**O**	**ME-SA**	**Ind-2001**	**Ind-2001e (Ind-2001BD 1)**	**https://doi.org/10.1038/s41598-023-36830-w**	**MGBL lab, University of Dhaka**
**BAN/DH/Ke-395/2020**	**OP320439.1**	**O**	**ME-SA**	**Ind-2001**	**Ind-2001e (Ind-2001BD 1)**	**https://doi.org/10.1038/s41598-023-36830-w**	**MGBL lab, University of Dhaka**
**BAN/TA/Mi-396/2020**	**OP271696.1**	**O**	**ME-SA**	**Ind-2001**	**Ind-2001e (Ind-2001BD 1)**	**https://doi.org/10.1038/s41598-023-36830-w**	**MGBL lab, University of Dhaka**
**BAN/DH/Sa-400/2020**	**OP320440.1**	**O**	**ME-SA**	**Ind-2001**	**Ind-2001e (Ind-**	**https://doi.org/10.1038/s41598-023-36830-w**	**MGBL lab, Universi**

					2001BD 1)		ty of Dhaka
**BAN/DH/Dh-410/2021**	**OP271697.1**	**O**	**ME-SA**	**Ind-2001**	**Ind-2001e (Ind-2001BD 1)**	https://doi.org/10.1038/s41598-023-36830-w	**MGBL lab, University of Dhaka**
**BAN/DH/Dh-416/2021**	**OP320441.1**	**O**	**ME-SA**	**Ind-2001**	**Ind-2001e (Ind-2001BD 1)**	https://doi.org/10.1038/s41598-023-36830-w	**MGBL lab, University of Dhaka**
**BAN/DH/Dh-417/2021**	**OP320442.1**	**O**	**ME-SA**	**Ind-2001**	**Ind-2001e (Ind-2001BD 1)**	https://doi.org/10.1038/s41598-023-36830-w	**MGBL lab, University of Dhaka**
**BAN/DH/Dh-418/2021**	**OP320443.1**	**O**	**ME-SA**	**Ind-2001**	**Ind-2001e (Ind-2001BD 1)**	https://doi.org/10.1038/s41598-023-36830-w	**MGBL lab, University of Dhaka**
**BAN/SA/Sa-423/2021**	**OP320444.1**	**O**	**ME-SA**	**Ind-2001**	**Ind-2001e (Ind-2001BD 1)**	https://doi.org/10.1038/s41598-023-36830-w	**MGBL lab, University of Dhaka**
**BAN/SA/Sa-426/2021**	**OP320445.1**	**O**	**ME-SA**	**Ind-2001**	**Ind-2001e (Ind-2001BD 1)**	https://doi.org/10.1038/s41598-023-36830-w	**MGBL lab, University of Dhaka**
**BAN/SA/Sa-431/2021**	**OP320446.1**	**O**	**ME-SA**	**Ind-2001**	**Ind-2001e (Ind-2001BD 1)**	https://doi.org/10.1038/s41598-023-36830-w	**MGBL lab, University of Dhaka**
**BAN/SA/Sa-432/2021**	**OP320447.1**	**O**	**ME-SA**	**Ind-2001**	**Ind-2001e (Ind-2001BD 1)**	https://doi.org/10.1038/s41598-023-36830-w	**MGBL lab, University of Dhaka**
**BAN/SA/Sa-433/2021**	**OP320448.1**	**O**	**ME-SA**	**Ind-2001**	**Ind-2001e (Ind-2001BD 1)**	https://doi.org/10.1038/s41598-023-36830-w	**MGBL lab, University of Dhaka**
**BAN/SA/Sa-440/2021**	**OP320449.1**	**O**	**ME-SA**	**Ind-2001**	**Ind-2001e (Ind-2001BD 1)**	https://doi.org/10.1038/s41598-023-36830-w	**MGBL lab, University of Dhaka**
**BAN/JH/Jh-454/2021**	**OP320450.1**	**O**	**ME-SA**	**Ind-2001**	**Ind-2001e (Ind-2001BD 1)**	https://doi.org/10.1038/s41598-023-36830-w	**MGBL lab, University of Dhaka**
**BAN/JH/Jh-455/2021**	**OP320451.1**	**O**	**ME-SA**	**Ind-2001**	**Ind-2001e (Ind-2001BD 1)**	https://doi.org/10.1038/s41598-023-36830-w	**MGBL lab, University of Dhaka**
**BAN/JH/Jh-457/2021**	**OP320452.1**	**O**	**ME-SA**	**Ind-2001**	**Ind-2001e (Ind-**	https://doi.org/10.1038/s41598-023-36830-w	**MGBL lab, Universi**

					2001BD 1)		ty of Dhaka
BAN/JH/Ka-461/2021	OP320453.1	O	ME-SA	Ind-2001	Ind-2001e (Ind-2001BD1)	https://doi.org/10.1038/s41598-023-36830-w	MGBL lab, University of Dhaka
BAN/JH/Ka-464/2021	OP320454.1	O	ME-SA	Ind-2001	Ind-2001e (Ind-2001BD1)	https://doi.org/10.1038/s41598-023-36830-w	MGBL lab, University of Dhaka
BAN LA Du-135 2013	KJ175181.1	O	ME-SA	Ind-2001	Ind-2001d	https://doi.org/10.1111/tbed.12834	MGBL lab, University of Dhaka
BAN/NA/Ha-156/2013	KF985189.1	O	ME-SA	Ind-2001	Ind-2001d	https://doi.org/10.1128/genomea.01253-13	MGBL lab, University of Dhaka
BAN JA Ma-180 2013	KJ175183.1	O	ME-SA	Ind-2001	Ind-2001d	https://doi.org/10.1111/tbed.12834	MGBL lab, University of Dhaka
BAN TA Dh-184 2013	KJ175184.1	O	ME-SA	Ind-2001	Ind-2001d	https://doi.org/10.1111/tbed.12834	MGBL lab, University of Dhaka
BAN TA Dh-185 2013	KJ175176.1	O	ME-SA	Ind-2001	Ind-2001d	https://doi.org/10.1111/tbed.12834	MGBL lab, University of Dhaka
BAN TA Dh-186 2013	KJ175185.1	O	ME-SA	Ind-2001	Ind-2001d	https://doi.org/10.1111/tbed.12834	MGBL lab, University of Dhaka
BAN RA Sa-189 2013	KJ175177.1	O	ME-SA	Ind-2001	Ind-2001d	https://doi.org/10.3329/bjm.v31i1.28464	MGBL lab, University of Dhaka
BAN/GA/Kk-191/2013	KY077602.1	O	ME-SA	Ind-2001	Ind-2001d	https://doi.org/10.1111/tbed.12834	MGBL lab, University of Dhaka
BAN/TA/Ma-200/2014	KY077604.1	O	ME-SA	Ind-2001	Ind-2001d	https://doi.org/10.1111/tbed.12834	MGBL lab, University of Dhaka
BAN/GA/Ka-212/2014	KY077605.1	O	ME-SA	Ind-2001	Ind-2001d	https://doi.org/10.1111/tbed.12834	MGBL lab, University of Dhaka
BAN/GA/Ka-213/2014	KY077606.1	O	ME-SA	Ind-2001	Ind-2001d	https://doi.org/10.1111/tbed.12834	MGBL lab, Universi

							ty of Dhaka
**BAN/GA/Ka-215/2015**	**KY077607.1**	**O**	**ME-SA**	**Ind-2001**	**Ind-2001d**	**https://doi.org/10.1111/tbed.12834**	**MGBL lab, University of Dhaka**
**BAN/DH/Dh-216/2015**	**KY077608.1**	**O**	**ME-SA**	**Ind-2001**	**Inter-sublineage**	**https://doi.org/10.1111/tbed.12834**	**MGBL lab, University of Dhaka**
**BAN/PA/Ch-228/2015**	**KY077609.1**	**O**	**ME-SA**	**Ind-2001**	**Ind-2001d**	**https://doi.org/10.1111/tbed.12834**	**MGBL lab, University of Dhaka**
**BAN LA Sa-137 2013**	**KJ175182.1**	**O**	**ME-SA**	**Ind-2001**	**Ind-2001BD2**	**https://doi.org/10.1111/tbed.12834**	**MGBL lab, University of Dhaka**
**BAN/BO/Na-161/2013**	**MK071699.1**	**O**	**ME-SA**	**Ind-2001**	**Ind-2001BD2**	**https://doi.org/10.1111/tbed.12834**	**MGBL lab, University of Dhaka**
**BAN/BO/Na-162/2013**	**KY077601.1**	**O**	**ME-SA**	**Ind-2001**	**Ind-2001BD2**	**https://doi.org/10.1111/tbed.12834**	**MGBL lab, University of Dhaka**
**BAN/MY/My-466/2021**	**OP320455.1**	**O**	**ME-SA**	**SA-2018**	**MYMBD21**	**https://doi.org/10.1038/s41598-023-36830-w**	**MGBL lab, University of Dhaka**
**BAN/MY/My-469/2021**	**OP320456.1**	**O**	**ME-SA**	**SA-2018**	**MYMBD21**	**https://doi.org/10.1038/s41598-023-36830-w**	**MGBL lab, University of Dhaka**
**BAN/MY/My-478/2021**	**OP320457.1**	**O**	**ME-SA**	**SA-2018**	**MYMBD21**	**https://doi.org/10.1038/s41598-023-36830-w**	**MGBL lab, University of Dhaka**
**BAN/MY/My-480/2021**	**OP320458.1**	**O**	**ME-SA**	**SA-2018**	**MYMBD21**	**https://doi.org/10.1038/s41598-023-36830-w**	**MGBL lab, University of Dhaka**
**BAN CH Ra-02 2012**	**KC795960.1**	**A**	**ASIA**	**G-VII**		**https://doi.org/10.1111/tbed.12113**	**MGBL lab, University of Dhaka**
**BAN CH Ra-08 2012**	**KC795949.1**	**A**	**ASIA**	**G-VII**		**https://doi.org/10.1111/tbed.12113**	**MGBL lab, University of Dhaka**
**BAN CH Ra-10 2012**	**KC795961.1**	**A**	**ASIA**	**G-VII**		**https://doi.org/10.1111/tbed.12113**	**MGBL lab, Universi**

							ty of Dhaka
**BAN CH Ra-13 2012**	**KC795962.1**	**A**	**ASIA**	**G-VII**		**https://doi.org/10.1111/tbed.12113**	**MGBL lab, University of Dhaka**
**BAN CH Ra-14 2012**	**KC795950.1**	**A**	**ASIA**	**G-VII**		**https://doi.org/10.1111/tbed.12113**	**MGBL lab, University of Dhaka**
**BAN CH Ra-15 2012**	**KC795951.1**	**A**	**ASIA**	**G-VII**		**https://doi.org/10.1111/tbed.12113**	**MGBL lab, University of Dhaka**
**BAN CH Ra-16 2012**	**KC795952.1**	**A**	**ASIA**	**G-VII**		**https://doi.org/10.1111/tbed.12113**	**MGBL lab, University of Dhaka**
**BAN CH Ra-18 2012**	**KC795953.1**	**A**	**ASIA**	**G-VII**		**https://doi.org/10.1111/tbed.12113**	**MGBL lab, University of Dhaka**
**BAN CH Ra-26 2012**	**KC795954.1**	**A**	**ASIA**	**G-VII**		**https://doi.org/10.1111/tbed.12113**	**MGBL lab, University of Dhaka**
**BAN CH Ra-28 2012**	**KC795955.1**	**A**	**ASIA**	**G-VII**		**https://doi.org/10.1111/tbed.12113**	**MGBL lab, University of Dhaka**
**BAN CH Ra-30 2012**	**KC795963.1**	**A**	**ASIA**	**G-VII**		**https://doi.org/10.1111/tbed.12113**	**MGBL lab, University of Dhaka**
**BAN CH Ra-31 2012**	**KC795964.1**	**A**	**ASIA**	**G-VII**		**https://doi.org/10.1111/tbed.12113**	**MGBL lab, University of Dhaka**
**BAN CH Ra-39 2012**	**KC795965.1**	**A**	**ASIA**	**G-VII**		**https://doi.org/10.1111/tbed.12113**	**MGBL lab, University of Dhaka**
**BAN GA To-02 2012**	**KC795948.1**	**A**	**ASIA**	**G-VII**		**https://doi.org/10.1111/tbed.12113**	**MGBL lab, University of Dhaka**
**BAN GA Sa-197 2013**	**KJ754939.1**	**A**	**ASIA**	**G-VII**		**https://doi.org/10.1128/genomeA.00506-14**	**MGBL lab, University of Dhaka**
**BAN/CH/Sa-302/2016**	**KY077629.1**	**A**	**ASIA**	**G-VII**		**https://doi.org/10.1111/tbed.12834**	**MGBL lab, Universi**

							ty of Dhaka
**BAN/CH/Sa-304/2016**	**KY077630.2**	**A**	**ASIA**	**G-VII**		**https://doi.org/10.1111/tbed.12834**	**MGBL lab, University of Dhaka**
**BAN/DH/Sa-307/2016**	**MN596870.1**	**A**	**ASIA**	**G-VII**		**This study**	**MGBL lab, University of Dhaka**
**BAN/DH/Dh-360/2019**	**OP320428.1**	**A**	**ASIA**	**G-VII**		**https://doi.org/10.1038/s41598-023-36830-w**	**MGBL lab, University of Dhaka**
**BAN/MA/Ma-362/2019**	**OP320429.1**	**A**	**ASIA**	**G-VII**		**https://doi.org/10.1038/s41598-023-36830-w**	**MGBL lab, University of Dhaka**
**BAN/CH/Ch-381/2020**	**OP320435.1**	**A**	**ASIA**	**G-VII**		**https://doi.org/10.1038/s41598-023-36830-w**	**MGBL lab, University of Dhaka**
**BAN/CH/Ch-386/2020**	**OP320436.1**	**A**	**ASIA**	**G-VII**		**https://doi.org/10.1038/s41598-023-36830-w**	**MGBL lab, University of Dhaka**
**BAN/DH/Ke-391/2020**	**OP320437.1**	**A**	**ASIA**	**G-VII**		**https://doi.org/10.1038/s41598-023-36830-w**	**MGBL lab, University of Dhaka**
**BAN JE Mf-01 2012**	**KJ175170.1**	**Asia 1**	**ASIA**	**G-VIII**		**https://doi.org/10.1111/lam.12354**	**MGBL lab, University of Dhaka**
**BAN JE Mf-02 2012**	**KJ175171.1**	**Asia 1**	**ASIA**	**G-VIII**		**https://doi.org/10.1111/lam.12354**	**MGBL lab, University of Dhaka**
**BAN JE Mf-03 2012**	**KJ175172.1**	**Asia 1**	**ASIA**	**G-VIII**		**https://doi.org/10.1111/lam.12354**	**MGBL lab, University of Dhaka**
**BAN JE Mf-04 2012**	**KJ175173.1**	**Asia 1**	**ASIA**	**G-VIII**		**https://doi.org/10.1111/lam.12354**	**MGBL lab, University of Dhaka**
**BAN JE Mf-05 2012**	**KJ175174.1**	**Asia 1**	**ASIA**	**G-VIII**		**https://doi.org/10.1111/lam.12354**	**MGBL lab, University of Dhaka**
**BAN JE Mf-06 2012**	**KJ175175.1**	**Asia 1**	**ASIA**	**G-VIII**		**https://doi.org/10.1111/lam.12354**	**MGBL lab, Universi**

							**ty of Dhaka**
**BAN/TA/Ma-167/2013**	**MF782478.1**	**Asia 1**	**ASIA**	**G-VIII**		**https://doi.org/10.1111/lam.12354**	**MGBL lab, University of Dhaka**
**BAN GA Sr-187 2013**	**KJ175186.1**	**Asia 1**	**ASIA**	**G-VIII**		**https://doi.org/10.1111/lam.12354**	**MGBL lab, University of Dhaka**
**BAN/DH/Sa-318/2018**	**MH457186.1**	**Asia 1**	**ASIA**	**G-IX**		**https://doi.org/10.1111/tbed.13381**	**MGBL lab, University of Dhaka**
**BAN/DH/Sa-319/2018**	**MH457187.1**	**Asia 1**	**ASIA**	**G-IX**		**https://doi.org/10.1111/tbed.13381**	**MGBL lab, University of Dhaka**
**BAN/BR/Sa-321/2018**	**MN447113.1**	**Asia 1**	**ASIA**	**G-IX**		**https://doi.org/10.1111/tbed.13381**	**MGBL lab, University of Dhaka**
**BAN/DH/Sh-323/2018**	**MN447114.1**	**Asia 1**	**ASIA**	**G-IX**		**https://doi.org/10.1111/tbed.13381**	**MGBL lab, University of Dhaka**
**BD Gh 2 2013**	**KT037119.1**	**O**	**ME-SA**	**Ind-2001**	**Ind-2001d**	**https://doi.org/10.1111/tbed.12834**	**Bangladesh Agricultural University**
**BD SI 6 2013**	**KT037120.1**	**O**	**ME-SA**	**Ind-2001**	**Ind-2001d**	**https://doi.org/10.1111/tbed.12834**	**Bangladesh Agricultural University**
**O/BAN/BLRI/450.1/2018**	**MT316588.1**	**O**	**ME-SA**	**Ind-2001**	**Ind-2001e (Ind-2001BD1)**	**https://doi.org/10.4314/ovj.v10i3.14**	**Bangladesh Livestock Research Institute (BLRI)**
**O/BAN/BLRI/450.3/2018**	**MT316589.1**	**O**	**ME-SA**	**Ind-2001**	**Ind-2001e (Ind-2001BD1)**	**https://doi.org/10.4314/ovj.v10i3.14**	**Bangladesh Livestock Research Institute (BLRI)**
**BD BAU ML3 2013**	**KT982204.1**	**A**	**ASIA**	**G-VII**		**https://doi.org/10.5455/javar.2020.g429**	**Bangladesh Agricultural University**

**BD BAU ML4 2014**	**KT982205.1**	**A**	**ASIA**	**G-VII**		**https://doi.org/10.5455/javar.2020.g429**	**Bangladesh Agricultural University**
**BD SI 16 2013**	**KR869773.1**	**A**	**ASIA**	**G-VII**		**https://doi.org/10.5455/javar.2020.g429**	**Bangladesh Agricultural University**
**BD AH 10 2012**	**KY421678.1**	**A**	**ASIA**	**G-VII**		**GenBank database**	**Bangladesh Livestock Research Institute (BLRI)**
**BD BAU ML5 2013**	**KU159763.1**	**Asia 1**	**ASIA**	**G-VIII**		**https://doi.org/10.5455/javar.2020.g429**	**Bangladesh Agricultural University**
**BD BAU ML6 2013**	**KU159762.1**	**Asia 1**	**ASIA**	**G-VIII**		**https://doi.org/10.5455/javar.2020.g429**	**Bangladesh Agricultural University**
**BD SI 2 2013**	**KR869774.1**	**Asia 1**	**ASIA**	**G-VIII**		**https://doi.org/10.5455/javar.2020.g429**	**Bangladesh Agricultural University**
**BD_SI_5_2013**	**KT037118.1**	**O**	**ME-SA**	**unnamed**		**https://doi.org/10.1111/tbed.12834**	**Bangladesh Agricultural University**
**BD_BAU_ML1_2013**	**KT960948.1**	**O**	**ME-SA**	**unnamed**		**https://doi.org/10.1111/tbed.12834 https://doi.org/10.5455/javar.2020.g429**	**Bangladesh Agricultural University**
**BD_BAU_ML2_2013**	**KT982203.1**	**O**	**ME-SA**	**unnamed**		**https://doi.org/10.1111/tbed.12834 https://doi.org/10.5455/javar.2020.g429**	**Bangladesh Agricultural University**
**O/BAN/BLRI/450.2/2018**	**MT316587.1**	**O**	**ME-SA**	**unnamed**		**https://doi.org/10.4314/ovj.v10i3.14**	**Bangladesh Livestock Research Institute (BLRI)**

Supplementary Table S8 Amino acid substitutions in VP1 of FMDV serotypes reported in Bangladesh during 2012-21

**Year**	**Serotype**	**Amino Acid Substitution**
2012	Asia 1	A69V
2012	O	E138A
2012	A	G148E
2012	A	T22I
2012	A	Q23K
2012	A	V24I
2012	A	I42L
2012	A	V45T
2012	A	T48I
2012	A	E83D
2012	A	V143T
2012	A	M190L
2012	A	E194D
2012	A	A209T
2013	O	V24A
2013	O	T43A
2013	O	K45Q
2013	O	N100S
2013	O	A110E
2013	O	S139G
2013	O	N140G, D, A
2013	O	P142T
2013	O	A155V
2013	O	A156T
2013	O	P158T
2013	O	A170T
2013	O	K181T
2013	O	A183P
2013	O	T185P
2013	O	Y186H
2013	O	E198Q, D
2013	O	R200T
2013	O	H201P
2013	O	K202Q
2013	O	K204N
2013	O	A207P
2013	O	K210N
2013	O	L212F
2013	O	Q45K
2013	O	N46D
2013	O	A171T

2013	O	D197E
2013	A	V24I
2013	A	I35V
2013	A	I42L
2013	A	V45T, A
2013	A	T48I
2013	A	E83D
2013	A	V143T
2013	A	R168K
2013	A	M190L
2013	A	S196L
2013	A	L213P
2013	Asia 1	T4A
2013	Asia 1	V24T
2013	Asia 1	A44T
2013	Asia 1	L80V
2013	Asia 1	D96G
2013	Asia 1	H108Q
2013	Asia 1	E202K
2013	Asia 1	V210M
2014	A	V24I
2014	A	I42L
2014	A	V45T
2014	A	T48I
2014	A	E83D
2014	A	V143T
2014	A	M190L
2014	O	E198D
2015	O	G19R
2015	O	T30S
2015	O	Q45K
2015	O	N46D
2015	O	A156T
2015	O	A171T
2015	O	D197E
2015	O	H201R
2015	O	K45Q
2015	O	T68A
2015	O	E95V
2015	O	D99E
2015	O	N100S
2015	O	K135R
2015	O	E138G
2015	O	N140G, D
2015	O	P142T, A
2015	O	E198Q

2015	O	K204R
2016	O	T43I
2016	O	Q45K
2016	O	N46D
2016	O	V62E
2016	O	E138K
2016	O	A171T
2016	O	D197E,G
2016	A	G43V
2016	A	S46G
2016	A	A68G
2016	A	N108Y
2016	A	A110V
2016	A	V143A
2018	O	S33P
2018	O	F39I
2018	O	T43I
2018	O	Q45K
2018	O	N46D
2018	O	V62E
2018	O	A171T
2018	O	D197E
2018	Asia 1	T3A
2018	Asia 1	V24T
2018	Asia 1	T43N
2018	Asia 1	A44E
2018	Asia 1	N47S
2018	Asia 1	I48T
2018	Asia 1	T50V
2018	Asia 1	D96T
2018	Asia 1	H108Q
2018	Asia 1	M146L
2018	Asia 1	M211L
2019	O	V15L
2019	O	E16R
2019	O	N17T
2019	O	G19R, P
2019	O	G20R
2019	O	K41I
2019	O	T43I
2019	O	Q45K
2019	O	N46D
2019	O	D52K
2019	O	T60N
2019	O	E138K
2019	O	A140T

2019	O	T142A
2019	O	A170S
2019	O	A171T
2019	O	D197E
2019	A	I42L
2019	A	G43V
2019	A	V45T
2019	A	R142H
2019	A	V143T
2019	A	E194K
2019	A	Q198R
2020	O	Q45K
2020	O	N46D
2020	O	K135R
2020	O	A171T
2020	O	V173G
2020	O	K181E
2020	O	D197E
2020	O	K204R
2020	O	L213F
2020	A	G19R
2020	A	N44D
2020	A	V45L
2020	A	S46N
2020	A	E83D
2020	A	N134S
2020	A	R142H
2020	A	V143T
2020	A	R168K
2020	A	M190L
2020	A	S196L
2021	O	Q45K
2021	O	N46D
2021	O	L76I
2021	O	K135R
2021	O	A171T
2021	O	D197E
